# The stromal vascular fraction mitigates bleomycin-induced skin fibrosis in mice by modulating vascular lesions and secreting antifibrotic factors at different stages of disease

**DOI:** 10.1186/s13287-025-04470-8

**Published:** 2025-07-01

**Authors:** Ziyu Wang, Mengqi Shi, Zonghao Liu, Yahui Chen, Xiangguang Shi, Jiucun Wang

**Affiliations:** 1 State Key Laboratory of Genetics and Development of Complex Phenotypes, Fudan University, Shanghai, China; 2 Human Phenome Institute, Fudan University, Shanghai, China; 3 Department of Dermatology, Huashan Hospital, Fudan University, Shanghai, China; 4 Department of Dermatology, Department of Allergy and Immunology, Research Center of Allergy and Diseases, Huashan Hospital, Fudan University, Shanghai, China; 5 Research Unit of Dissecting the Population Genetics and Developing New Technologies for Treatment and Prevention of Skin Phenotypes and Dermatological Diseases (2019RU058), Chinese Academy of Medical Sciences, Shanghai, China

**Keywords:** Systemic sclerosis, Stromal vascular fraction, Skin fibrosis, Bleomycin-induced skin fibrosis

## Abstract

**Background:**

Systemic sclerosis (SSc) is a chronic autoimmune disease characterized by fibrosis of the skin and internal organs, leading to significant morbidity and reduced quality of life. Despite ongoing research, the underlying pathogenesis of SSc remains unclear, and treatment options are limited. Stromal vascular fraction (SVF), a naturally occurring cell population that includes mesenchymal stem cells (MSCs), has emerged as a potential therapeutic agent for various fibrotic diseases. This study aimed to investigate the therapeutic effects and underlying mechanisms of SVF in a bleomycin-induced mouse model of skin fibrosis.

**Methods:**

SVF was isolated from the inguinal adipose tissue of C57BL/6 mice and administered subcutaneously or intradermally at different disease stages to assess its impact on skin fibrosis. Histological analyses were performed to evaluate dermal thickness and collagen deposition. In vivo imaging and immunofluorescence were used to track the retention of SVF within fibrotic tissue over time, particularly in the subcutaneous layer. Flow cytometry and immunofluorescence were employed to examine cutaneous vascular pathology and the secretion of antifibrotic factors, such as hepatocyte growth factor (HGF) and basic fibroblast growth factor (FGF-2). Finally, we investigated the contribution of major SVF subsets to cutaneous fibrosis and the mechanisms by which these subsets mediate therapeutic effects.

**Results:**

SVF significantly attenuated skin fibrosis in both early and late stages of disease, as evidenced by reduced dermal thickness and collagen deposition. Notably, SVF showed prolonged retention in fibrotic tissues—especially in the subcutaneous layer—for at least 18 days post-injection, with antifibrotic effects primarily mediated through paracrine mechanisms. In early-stage fibrosis, SVF inhibited endothelial–mesenchymal transition and mitigated skin vascular damage. In late-stage fibrosis, SVF continued to secrete antifibrotic factors, including HGF and FGF-2. Subsequent analyses identified the CD45-negative subset of SVF as a key regulator of skin fibrosis.

**Conclusion:**

SVF, particularly its CD45-negative subset, holds considerable promise for the treatment of SSc-associated skin fibrosis. These findings suggest that SVF-based therapies could be effective in managing fibrosis-related diseases and offer valuable insights for future clinical applications.

## Background

Systemic sclerosis (SSc), also known as scleroderma, is a chronic autoimmune disease characterized by widespread vascular abnormalities, immune activation, and progressive fibrosis affecting the skin and internal organs [1]. Among its clinical manifestations, skin fibrosis is particularly prominent, significantly impairing patients’ daily activities and quality of life. The pathogenesis of SSc is complex and involves multifactorial interactions among immune dysfunction, endothelial damage, and fibroblast activation [2, 3].

Current treatment strategies for SSc include immunosuppressive agents, such as cyclophosphamide and mycophenolate mofetil, as well as antifibrotic agents like nintedanib [4]. Although these treatments can slow disease progression, their ability to reverse established fibrosis is limited, and many are associated with substantial adverse effects. In recent years, cell-based therapies have attracted increasing attention as potential treatments for fibrotic diseases. Among these, mesenchymal stem cells (MSCs) have become a research focus due to their immunomodulatory, proangiogenic, and antifibrotic properties [5, 6]. Preclinical studies have demonstrated that MSCs can significantly reduce skin thickness, collagen deposition, vasculopathy, and immune dysfunction [7, 8]. Clinical trials have further shown that intravenous infusion or topical injections of MSCs can decrease the Modified Rodnan Skin Score and improve skin pliability [9, 10]. However, despite their promising therapeutic potential, the clinical application of MSCs is limited by challenges such as large-scale expansion, decreased efficacy during long-term culture, safety concerns, and the high time and cost required for MSC preparation [11]. Moreover, MSCs are primarily recognized for their immunoregulatory roles, but their ability to substantially modulate angiogenesis remains limited [12].

Stromal vascular fraction (SVF), a primary source of MSCs, has been extensively investigated as a naturally occurring MSC-containing cell population [13]. SVF is obtained through enzymatic digestion of adipose tissue and comprises a heterogeneous mix of MSCs, endothelial cells, immune cells, and vascular progenitor cells [14]. Unlike MSCs, which are predominantly expanded in vitro, SVF retains unexpanded MSCs along with their intrinsic microenvironment and paracrine functions. Additionally, SVF can be isolated in a single procedure, allowing immediate autologous use without complex in vitro expansion. This decreases production costs and maximizes cell viability and function, making SVF particularly advantageous in clinical settings [15]. As a result, SVF is considered an optimal choice for MSC-based therapy and has shown considerable potential in treating fibrosis-related diseases.

Clinical studies have demonstrated that SVF exhibits a favorable safety profile for the treatment of pulmonary fibrosis [16], highlighting its promise for clinical applications. Furthermore, multiple clinical trials have reported that SVF effectively alleviates vocal cord scarring [17], and myositis-related fibrosis [18]. In addition, clinical trials focused on scleroderma-related hand fibrosis have shown that SVF can significantly improve vascular lesions and reduce fibrosis [19–21]. These findings suggest that SVF promotes angiogenesis, diminishes inflammation, and suppresses fibrosis. Nonetheless, despite these positive indications, both the efficacy and underlying mechanisms of SVF in SSc-associated skin fibrosis have yet to be clearly elucidated.

This study was designed to evaluate the therapeutic effects of SVF on scleroderma-associated skin fibrosis and to investigate its mechanisms of action using a bleomycin-induced mouse model. This model is widely employed to simulate key features of SSc-related skin fibrosis. Our research aimed to provide crucial insights into how SVF mitigates skin fibrosis and to lay the groundwork for its potential clinical application in SSc treatment.

## Methods

### Isolation and identification of SVF

All animal experiments were conducted in accordance with the guidelines approved by the Institutional Animal Care and Use Committee of Fudan University (Approval No. IDM2022034a). Inguinal adipose tissue from C57BL/6 mice was harvested and cut into 1 mm³ pieces, followed by enzymatic digestion with 1 WU/mL Celase (Cytori Therapeutics, USA). After digestion, the tissue was centrifuged to separate the adipocyte layer from the SVF cell layer. The lower SVF cell layer was collected, passed through a 70 μm cell strainer, and subjected to erythrocyte lysis to obtain the final SVF cells. SVF subpopulations were identified via flow cytometry (LSR Fortessa; BD Biosciences, USA) using two antibody panels: Panel 1: FVD-eF780, CD45-BV421, CD44-PE, CD34-AF488, CD31-AF647; Panel 2: FVD-eF780, CD45-BV421, F4/80-PE, CD11b-BV510, CD3e-FITC, B220-PECy7, Ly6C/G-BV786. FVD was purchased from Thermo Fisher (USA), and all other flow cytometry antibodies were obtained from BD Biosciences (USA). To characterize adipose-derived MSCs within SVF, cells were cultured to passage 3, and osteogenic, chondrogenic, and adipogenic differentiation were performed according to the manufacturer’s protocol for the Mouse Adipose MSCs Induced Differentiation Kit (OriCell, China).

### Bleomycin-induced skin fibrosis model and SVF administration

Eight-week-old female C57BL/6 mice (SPF Biotechnology, China) were used for the bleomycin-induced skin fibrosis model. Mice received subcutaneous injections of 100 µL of bleomycin (1 mg/mL; MCE, China) into alternating corners of a designated injection area daily for 21 consecutive days. The healthy control group received saline injections. SVF (5 × 10^5^ cells per mouse) was administered either subcutaneously or intradermally. Mice were randomly assigned to experimental groups using a random number generator to minimize selection bias. Sample size was calculated using one-way ANOVA to ensure statistical rigor. Based on preliminary data, effect size was estimated, and group sizes were determined using G*Power 3.1. Following the 3R principles (Replacement, Reduction, and Refinement), we minimized animal use while ensuring statistical validity. Specific sample sizes (n) are provided in the figure legends. All mice that successfully received subcutaneous bleomycin or saline injections were included in the study. Mice were excluded if the injection failed due to needle dislodgement or other technical issues preventing successful administration of bleomycin or saline. Each mice was handled by three researchers. The first researcher, who knew the treatment group allocation, administered the treatment based on randomization. The other two researchers, blinded to the group allocation, performed the surgeries and evaluated the outcomes. At the end of the experiment, mice were euthanized using carbon dioxide (CO_2_) asphyxiation, following established ethical guidelines for animal research. The work has been reported in line with the ARRIVE guidelines 2.0.

### Retention of SVF in mouse skin

To investigate the retention of SVF in mouse skin, we first included a bleomycin-only (BLM) group to account for potential autofluorescence in the Dir detection channel. Two experimental groups were established for comparison: the BLM + Dir-labelled SVF group, in which Dir-labelled SVF was administered subcutaneously on day 3 following bleomycin induction, and the CON + Dir-labelled SVF group, in which Dir-labelled SVF was injected on day 3 after saline administration instead of bleomycin. These groups were used to compare SVF retention under fibrotic versus non-fibrotic conditions. Subsequently, skin sections from the BLM + Dir-labelled SVF group were collected on days 7, 14, and 21 post bleomycin modeling and subjected to immunofluorescence staining for Dir and CD45 to assess the overall retention of Dir-labelled SVF cells. In parallel, co-staining for Dir and CD31 was performed on the same time points to determine the spatial distribution of CD31 positive subpopulations within the retained Dir-labelled SVF cells.

### Histological and immunofluorescence evaluation

Skin samples were fixed in 4% paraformaldehyde for paraffin embedding or cryopreserved in liquid nitrogen for frozen sections. Histological evaluation was performed using hematoxylin and eosin (HE) and Masson’s trichrome staining. Inflammatory infiltration was scored, and collagen volume was quantified using ImageJ software (version 1.54 m; National Institutes of Health, USA). For immunofluorescence staining on paraffin sections, DAPI (Sigma, USA), α-SMA antibody (Sigma, USA), and CD31 antibody (AiFang Biological, China) were used. For frozen sections, DAPI (Sigma, USA), CD31 antibody (Abcam, USA), CD45 antibody (Abcam, USA), HGF antibody (Proteintech, China), and FGF-2 antibody (Proteintech, China) were employed. Image acquisition and analysis were conducted with a Stellaris 5 confocal laser microscope (Leica, Germany) using LAS X software.

### SVF labeling and live imaging

SVF cells were labeled with 7.5 μm Dir (MCE, China) by co-incubation at 37 °C for 15 min. Labeling efficiency was confirmed via flow cytometric analysis. For in vivo imaging, 3 × 10^6^ Dir-labeled SVF cells were administered subcutaneously to mice. During imaging, mice were initially anesthetized with 2.5% isoflurane (RWD Life Science, China) in oxygen at a flow rate of 1.0 L/min in an induction chamber. Anesthesia was then maintained with 2% isoflurane at a flow rate of 0.6 L/min *via* a nose cone throughout the procedure. An IVIS Lumina system (PerkinElmer, Germany) and Living Image software were used for in vivo fluorescence imaging and data processing.

### Identification of SVF subpopulations in mouse skin

A digestive solution was prepared by dissolving Collagenase I (2 mg/mL; Sigma, USA), Collagenase IV (2 mg/mL; Sigma, USA), Dispase II (2 U/mL; Sigma, USA), and DNase I (1 mg/mL; Roche, USA) in PBS. Mouse skin was minced into small pieces, transferred to the digestive solution, and incubated in a tissue-shaking digester at 37 °C for 1 h. The resulting solution was filtered through a 70 μm strainer, centrifuged, and treated with erythrocyte lysis buffer to obtain single-cell suspensions. Dir-positive SVF subpopulations were identified by flow cytometry using FVD-eF455UV, CD45-BV421, CD34-AF488, CD31-AF647, CD3e-BUV496, B220-PECy7, F4/80-PE, CD11b-BV510, and Ly6C/G-BV786. FVD was purchased from Thermo Fisher (USA), and all other antibodies were obtained from BD Biosciences (USA).

### Quantitative real-time PCR (qPCR)

Total RNA was isolated from mouse skin samples using TRIzol (Life Sciences, USA) and reverse-transcribed to cDNA with the PrimeScript RT Master Mix kit (TaKaRa, Japan). The expression levels of endothelial inflammation-associated genes (*P-selection*,* E-selection*,* VCAM1*) were determined by qPCR using Power SYBR Green Master Mix (Life Sciences, USA). mRNA levels of the target genes were normalized to β-actin mRNA. Primer sequences are provided in Table 1.

**Table 1:** Primer sequences for quantitative Real-time PCR

Name	5’-3’	Sequence
*P-selection*	Forward Reverse	GTCTGTCCCGTCACTGGATAC TCCTCTCTTACCGGGTTACCA
*E-selection*	Forward Reverse	ATGCCTCGCGCTTTCTCTC GTAGTCCCGCTGACAGTATGC
*Vcam1*	Forward Reverse	AGTTGGGGATTCGGTTGTTCT CCCCTCATTCCTTACCACCC
*β-actin*	Forward Reverse	TGTCCACCTTCCAGCAGATGT AGCTCAGTAACAGTCCGCCTAG

### Statistical analysis

Data normality was assessed with the Shapiro–Wilk test. Normally distributed data were analyzed by one-way ANOVA for single-factor comparisons or two-way ANOVA for multifactor analyses, followed by Tukey’s post-hoc test for pairwise comparisons. For non-normally distributed data, the Kruskal–Wallis test was employed, followed by Dunn’s multiple comparisons test with Bonferroni correction to control for type I errors. All statistical analyses and data visualizations were performed using PRISM (version 10.0; GraphPad Software, USA).

## Results

### SVF contains multiple cell subpopulations, including adipose-derived progenitor/stromal cells

**Fig. 1:**
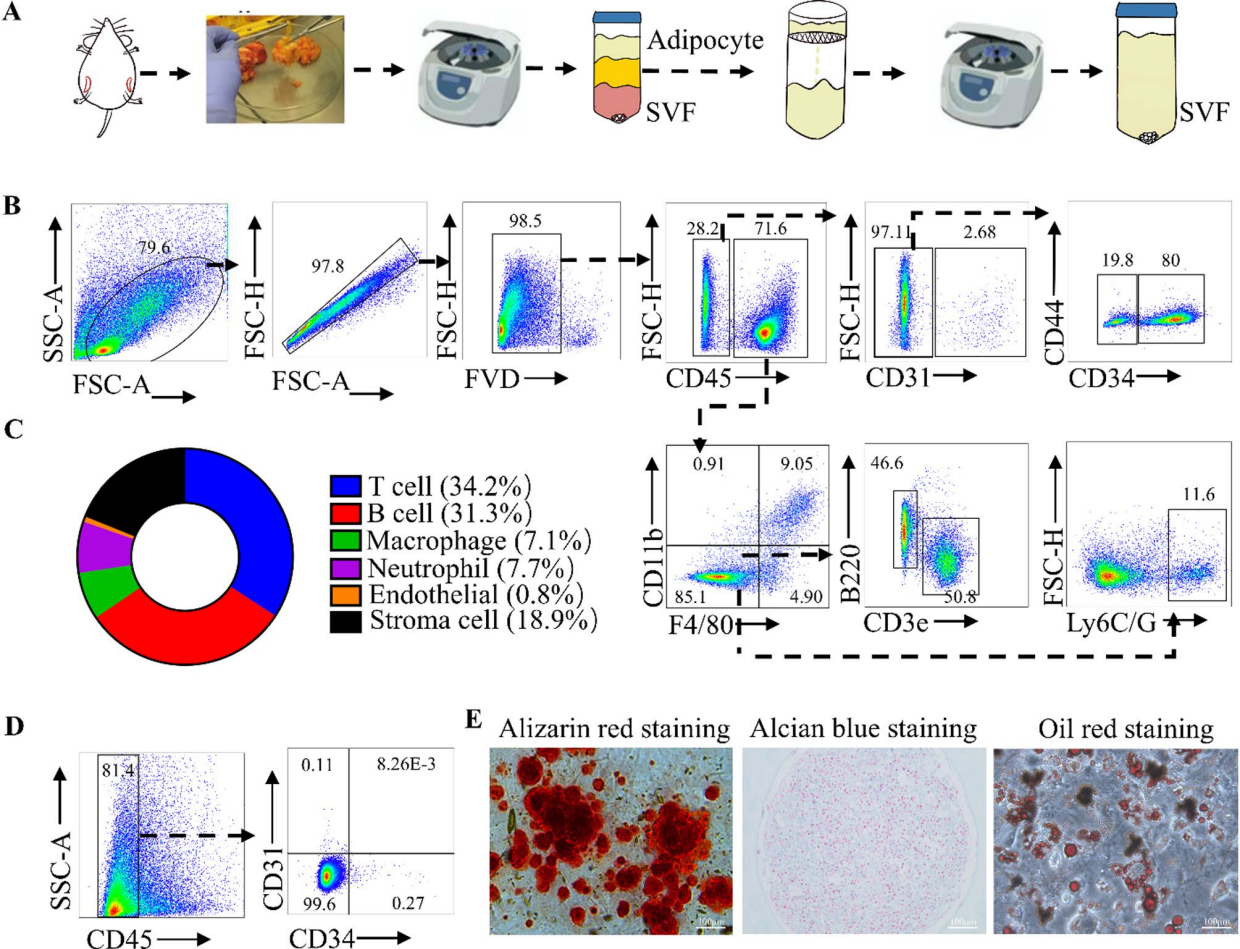
SVF contains multiple cell subpopulations, including adipose-derived progenitor cells. We collected mouse-derived inguinal fat and obtained SVF by enzymatic digestion (**A**) and identified each cell subpopulation (**B**) and its ratio (**C**) by flow cytometry. SVF was cultured to the third passage to obtain adipose-derived MSCs, which were assessed by flow cytometry (**D**) and characterized for their osteogenic, chondrogenic, and adipogenic differentiation capacities (**E**)

By enzymatically digesting mouse inguinal adipose tissue, we successfully isolated and obtained SVF (Fig. 1A). Flow cytometry indicated that SVF contained various cell populations, including T cells (34.2%), B cells (31.3%), macrophages (7.1%), neutrophils (7.7%), stromal cells (18.9%), and endothelial cells (0.8%) (Fig. 1B, C). As a naturally occurring source of MSCs, SVF is considered an optimal choice for MSC-based cell therapy. Accordingly, we investigated whether the isolated SVF contained functional MSCs. After adherent culture, SVF cells exhibited MSC-like characteristics, lacked expression of the immune cell marker CD45 and the hematopoietic stem cell marker CD34 (Fig. 1D), and demonstrated the capacity for osteogenic, chondrogenic, and adipogenic differentiation (Fig. 1E).

### Both early and late administration of SVF effectively inhibits bleomycin-induced skin fibrosis in mice

**Fig. 2:**
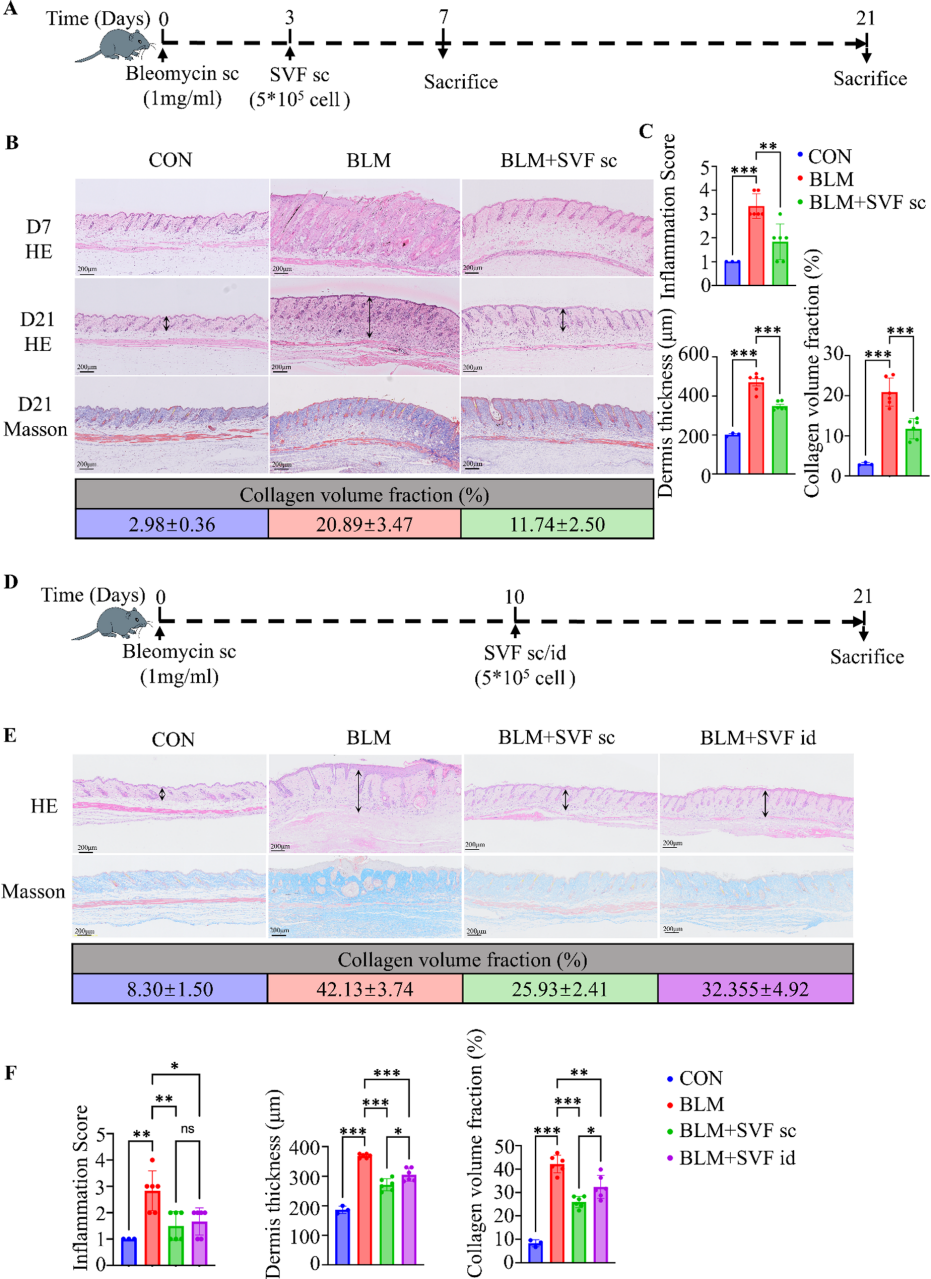
Early and late SVF administration effectively alleviates bleomycin-induced skin fibrosis. **A**: Subcutaneous injection of bleomycin-induced skin fibrosis using C57BL/6 mice, early subcutaneous administration of SVF on day 3, and assayed on days 7 and 21 post bleomycin modeling. sc: Subcutaneous Injection. **B**: Representative hematoxylin and eosin (HE) and Masson’s trichrome images of skin sections from early SVF administration groups at days 7 and 21. Collagen volume fraction (%) is presented as mean ± SD (control group, *n* = 3; all other groups, *n* = 6). **C**: Histograms of inflammatory cell infiltration, dermal thickness, and collagen volume for early SVF administration (control group, *n* = 3; all other groups, *n* = 6), analyzed by one-way ANOVA. **D**: Subcutaneous injection of bleomycin using C57BL/6 mice to induce skin fibrosis, late administration of SVF on day 10, and assay on day 21 post bleomycin modeling. id: Intradermal Injection. **E**: Representative HE and Masson’s trichrome images of skin sections at day 21 for late SVF administration. Collagen volume fraction (%) is shown as mean ± SD (control group, *n* = 3; all other groups, *n* = 6). **F**: Histograms of inflammatory cell infiltration, dermal thickness, and collagen volume for late SVF administration (control group, *n* = 3; all other groups, *n* = 6), analyzed by two-way ANOVA. Data are presented as mean ± SD. *p < 0.05, **p < 0.01, ***p < 0.001

To examine the therapeutic effect of SVF on skin fibrosis, we employed a bleomycin-induced skin fibrosis model in C57BL/6 mice and administered SVF in the early phase (day 3 post bleomycin modeling) (Fig. 2A). Bleomycin triggered inflammatory cell infiltration in the early stage of disease and progressed to fibrosis at a later stage. Notably, subcutaneous administration of SVF during the early stage significantly reduced inflammatory infiltration scores, dermal thickness, and collagen deposition at the fibrotic stage (Fig. 2B, C). Building on the prophylactic effect of early subcutaneous SVF administration, we further investigated its therapeutic potential at a later time point (day 10 post bleomycin modeling). Because subcutaneous and intradermal administration are both used clinically for treating skin diseases, we compared their efficacy in mitigating bleomycin-induced skin fibrosis (Fig. 2D). The results showed that at the advanced stage, both subcutaneous and intradermal administration of SVF effectively reduced inflammatory infiltration, dermal thickness, and collagen deposition, with subcutaneous administration demonstrating superior efficacy in reducing dermal thickness and collagen content (Fig. 2E, F). These findings suggest that SVF exerts significant antifibrotic effects when administered during both the early and late stages of fibrosis. Moreover, the mode of administration influences therapeutic efficacy, with subcutaneous injection showing greater effectiveness in mitigating bleomycin-induced skin fibrosis in mice. This observation provides valuable insights for optimizing clinical administration strategies for SVF.

### SVF exhibits long-term retention in fibrotic skin

**Fig. 3:**
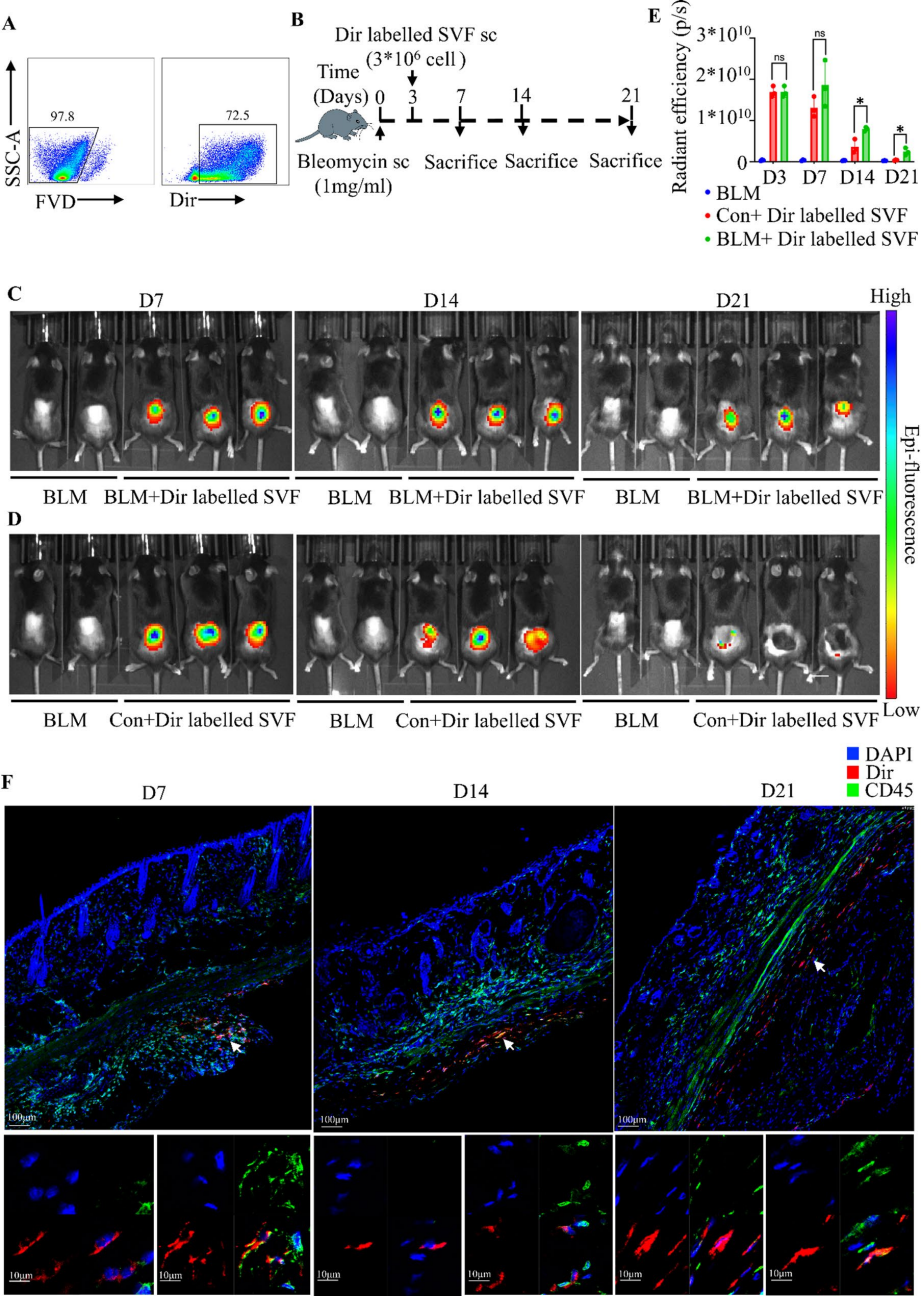
Subcutaneous administration of SVF enables long-term retention. **A**: Flow cytometry confirming the proportion of Dir-positive SVF following Dir labeling. **B**: Experimental design in which skin fibrosis was induced by subcutaneous injection of bleomycin or saline in C57BL/6 mice, followed by early sc administration of Dir-labeled SVF on day 3 and analysis on days 7, 14, and 21 post bleomycin modeling. **C**: Representative in vivo images of Dir fluorescence in different groups of mice at different time points, BLM group: bleomycin modelling only. BLM + Dir labelled SVF group: Dir-labelled SVF administered subcutaneously on day 3 after bleomycin modelling. **D**: Representative in vivo images of Dir fluorescence in different groups of mice at different time points, BLM group: bleomycin modelling only. CON + Dir labelled SVF group: Dir-labelled SVF given subcutaneously on day 3 after modelling with saline instead of bleomycin. **E**: Histograms showing relative fluorescence intensity at the site of subcutaneous administration (ROI) in each group (*n* = 3). Statistical analyses were performed using one-way ANOVA. **F**: Representative immunofluorescence images of mouse skin at different time points in the BLM + Dir labelled SVF group. Data are shown as mean ± SD. *p < 0.05

Given the significant antifibrotic effects of SVF at various stages and via different administration routes, we next investigated its retention in fibrotic skin to elucidate the persistence of its therapeutic efficacy and underlying mechanisms. SVF was labeled with the live-cell dye Dir (Fig. 3A) and administered subcutaneously at the early inflammatory stage (day 3 post bleomycin modeling). The retention of Dir-labeled SVF was subsequently monitored via in vivo fluorescence imaging and immunofluorescence analysis of skin sections at multiple time points post-SVF injection (Fig. 3B).

In vivo fluorescence imaging revealed that SVF remained localized at the injection site with sustained retention on days 4, 11, and 18 post-SVF injection (Fig. 3C). Notably, compared with Con + Dir labelled SVF group, SVF retention in the BLM + Dir labelled SVF group was prolonged, with higher fluorescence intensity (Fig. 3D, E), suggesting that SVF may preferentially remain in fibrotic tissues. Immunofluorescence of skin sections showed that SVF persisted in the subcutaneous region without migrating into the dermis over time (Fig. 3F), indicating that SVF likely exerts therapeutic effects predominantly via paracrine mechanisms rather than by direct transdifferentiation into dermal cells. Further analysis revealed that both CD45-negative and CD45-positive subpopulations of SVF were retained for extended periods. Over time, the nuclear morphology of SVF cells shifted from a round shape to an elongated form, suggesting dynamic changes in the dominant cell types within SVF (Fig. 3F).

These results demonstrate that SVF exhibits robust colonization in fibrotic skin and persists in the subcutaneous tissue over an extended timeframe. The therapeutic effects of SVF are likely mediated through paracrine pathways rather than direct differentiation into dermal cells. Moreover, the long-term retention of both CD45-negative and CD45-positive subpopulations, coupled with observed changes in nuclear morphology, indicates that the cellular composition of SVF may undergo time-dependent adaptations, which could be critical for maintaining sustained antifibrotic efficacy.

### SVF mitigates dermal vasculopathy in the early stages of skin fibrosis

**Fig. 4:**
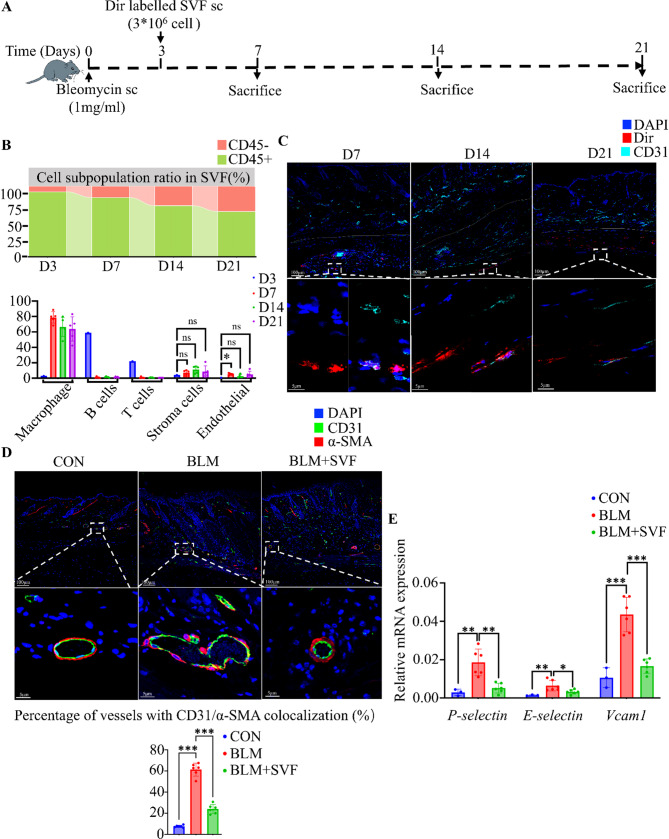
SVF ameliorates dermal vasculopathy in the early stages of skin fibrosis. **A**: C57BL/6 mice were injected subcutaneously with bleomycin to induce skin fibrosis; Dir-labeled SVF was administered subcutaneously early on day 3, and skin samples were collected on days 7, 14, and 21 post bleomycin modeling. **B**: The proportion of each SVF cell type at different time points in different groups, D3 group: Dir-labelled SVF prior to subcutaneous injection (*n* = 1), D7 group: Dir-positive cells isolated from mouse skin on day 4 after subcutaneous injection of Dir-labelled SVF (*n* = 5). D14 group: Dir-positive cells isolated from mouse skin on day 11 after subcutaneous injection of Dir-labelled SVF (*n* = 5), D21 group: Dir-positive cells isolated from mouse skin on day 18 after subcutaneous injection of Dir-labelled SVF (*n* = 5), analyzed by one-way ANOVA. **C**: Representative immunofluorescence images of bleomycin-induced fibrotic mouse skin at different time points after Dir-labelled SVF administration. The white line denotes the dermal–subcutaneous interface. **D**: Representative immunofluorescence images of CD31/α-SMA co-localized vessels on day 7 post bleomycin modeling (*n* = 6). Statistical analyses were performed using one-way ANOVA. **E**: *P-selectin*,* E-selectin*,* VCAM-1* gene expression levels in day 7 mouse skin following bleomycin modeling (control group, *n* = 3; all other groups, *n* = 6), analyzed by one-way ANOVA. Data are presented as mean ± SD. *p < 0.05, **p < 0.01, ***p < 0.001

Having observed that SVF shows prolonged retention in fibrotic skin and targets fibrotic sites, we next explored its potential mechanism of action during the early inflammatory phase. Dir-labeled SVF was administered subcutaneously during this phase, and Dir-positive SVF cells retained in the tissue were isolated on days 4, 11, and 18 post-SVF injection to evaluate subpopulation retention via flow cytometry (Fig. 4A). The proportion of CD45-positive cells gradually declined over time, whereas the proportion of CD45-negative cells progressively increased (Fig. 4B). Notably, the fraction of endothelial cells in SVF rose significantly on day 4 post-SVF injection (Fig. 4B) and was localized within the subcutaneous region (Fig. 4C), suggesting that SVF-derived endothelial cells might mediate early vascular changes in bleomycin-induced skin fibrosis via paracrine effects. In the early stage of bleomycin-induced skin fibrosis, dermal vascular cells underwent substantial endothelial–mesenchymal transition (EndoMT), characterized by increased co-expression of CD31 and α-SMA and elevated expression of endothelial inflammation-associated genes (*P-selectin*, *E-selectin*, and *VCAM1*) in skin tissue. Following early subcutaneous SVF administration, both the proportion of blood vessels undergoing EndoMT in the dermis and the expression levels of *P-selectin*,* E-selectin*, and *VCAM1* in mouse skin were significantly reduced (Fig. 4D, E). These findings indicate that early SVF injection effectively inhibits bleomycin-induced EndoMT and reduces the expression of endothelium-associated inflammatory genes. Thus, SVF-derived endothelial cells likely contribute to vascular repair via paracrine mechanisms, improving the inflammatory microenvironment in the early phases of bleomycin-induced fibrosis and providing a potential mechanism by which early SVF administration may attenuate fibrotic progression.

### SVF secretes antifibrotic factors in the late stages of skin fibrosis

**Fig. 5:**
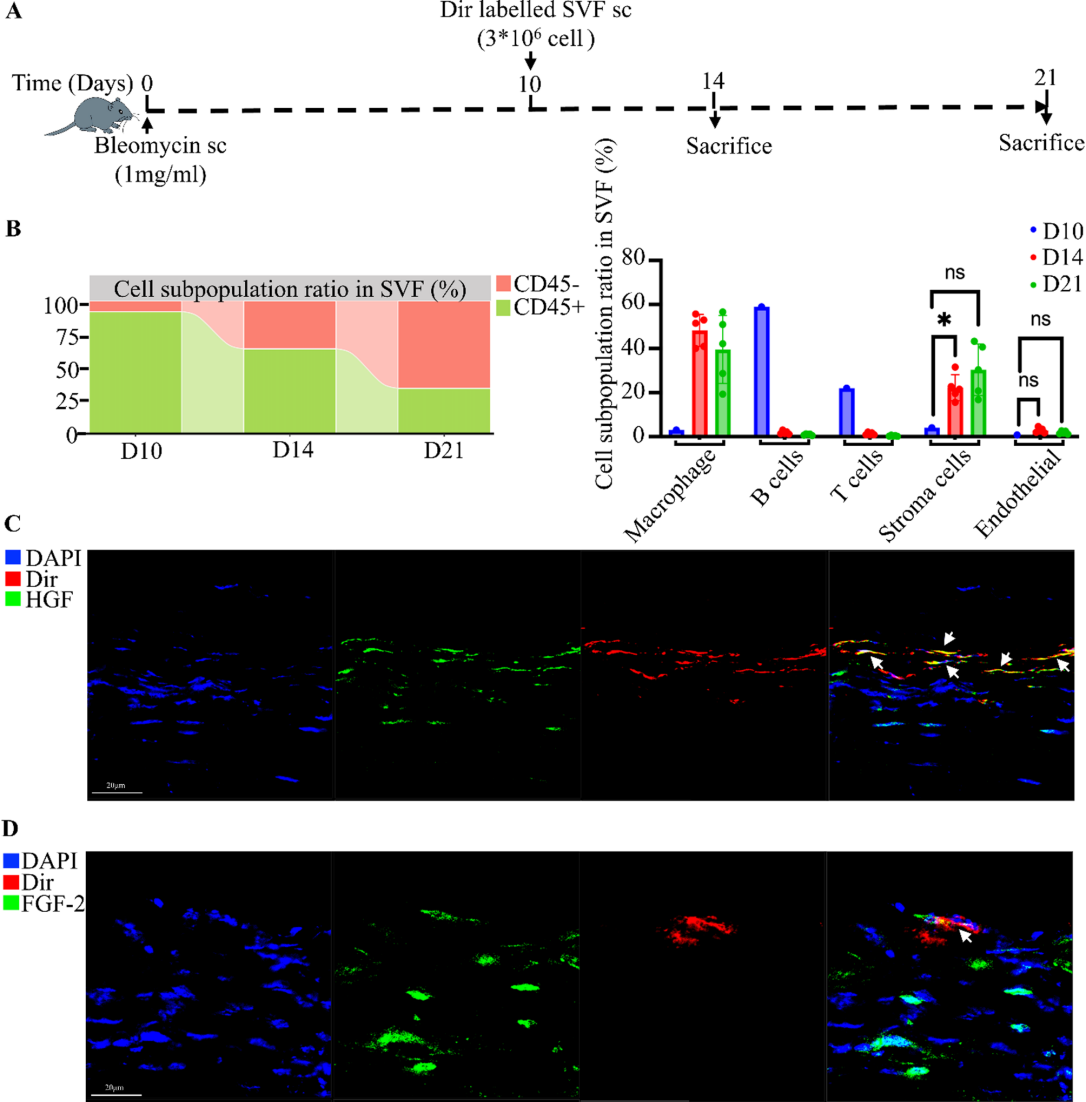
SVF can secrete antifibrotic factors in the late stages of skin fibrosis. **A**: Skin fibrosis was induced by subcutaneous injection of bleomycin in C57BL/6 mice; Dir-labeled SVF was administered subcutaneously late on day 10 and assayed on days 14 and 21 post bleomycin modeling. **B**: The proportion of each SVF subpopulation at different time points in different groups, D10 group: Dir-labelled SVF prior to subcutaneous injection (*n* = 1), D14 group: Dir-positive cells isolated from mouse skin on day 4 after subcutaneous injection of Dir-labelled SVF (*n* = 5). D21 group: Dir-positive cells isolated from mouse skin on day 11 after subcutaneous injection of Dir-labelled SVF (*n* = 5). Statistical analyses were performed by one-way ANOVA. **C**: Representative immunofluorescence staining for HGF in mouse skin at D21 group. **D**: Representative immunofluorescence staining for FGF-2 in mouse skin at D21 group. Data are presented as mean ± SD. *p < 0.05

Given that early SVF administration ameliorates vascular lesions and modulates the inflammatory microenvironment via paracrine effects mediated by endothelial cells, we next investigated SVF’s mechanism of action when administered during the late stage of fibrosis. Dir-labeled SVF was injected subcutaneously at the late fibrotic phase, and Dir-positive SVF was isolated on days 4 and 11 post- SVF injection. Flow cytometry was then used to analyze the retention of each SVF subpopulation (Fig. 5A). Results showed that the proportion of CD45-positive cells decreased over time, whereas the proportion of CD45-negative cells increased. Notably, the proportion of CD45-negative cells was higher in late administration than in early administration (Fig. 5B). Unlike early administration, where endothelial cells were predominant, late SVF injection did not cause significant changes in the proportion of endothelial cells. Instead, the stromal cell fraction rose markedly (Fig. 5B) and was mainly localized in the subcutaneous region. These findings suggest that stromal cells are the principal mediators of SVF’s antifibrotic effects during late administration, likely through paracrine mechanisms. In particular, hepatocyte growth factor (HGF) and basic FGF-2 have both been shown to reduce dermal thickness, collagen deposition, and scar formation in skin fibrosis models. Immunofluorescence analysis of late-stage fibrotic skin confirmed that stromal cells in SVF expressed the antifibrotic factors HGF (Fig. 5C) and FGF-2 (Fig. 5D). These observations indicate that stromal cells within SVF play a key paracrine role in mitigating skin fibrosis at later stages by secreting HGF and FGF-2, thereby reducing dermal thickness and collagen deposition.

### The CD45-negative subpopulation of SVF independently regulates skin fibrosis

Since SVF exerts notable antifibrotic effects during both early and advanced fibrosis—through endothelial and stromal cells, respectively—we next investigated whether the CD45-negative subpopulation of SVF could independently regulate skin fibrosis. This analysis was intended to clarify the significance of the CD45-negative fraction in SVF’s overall therapeutic action.

**Fig. 6:**
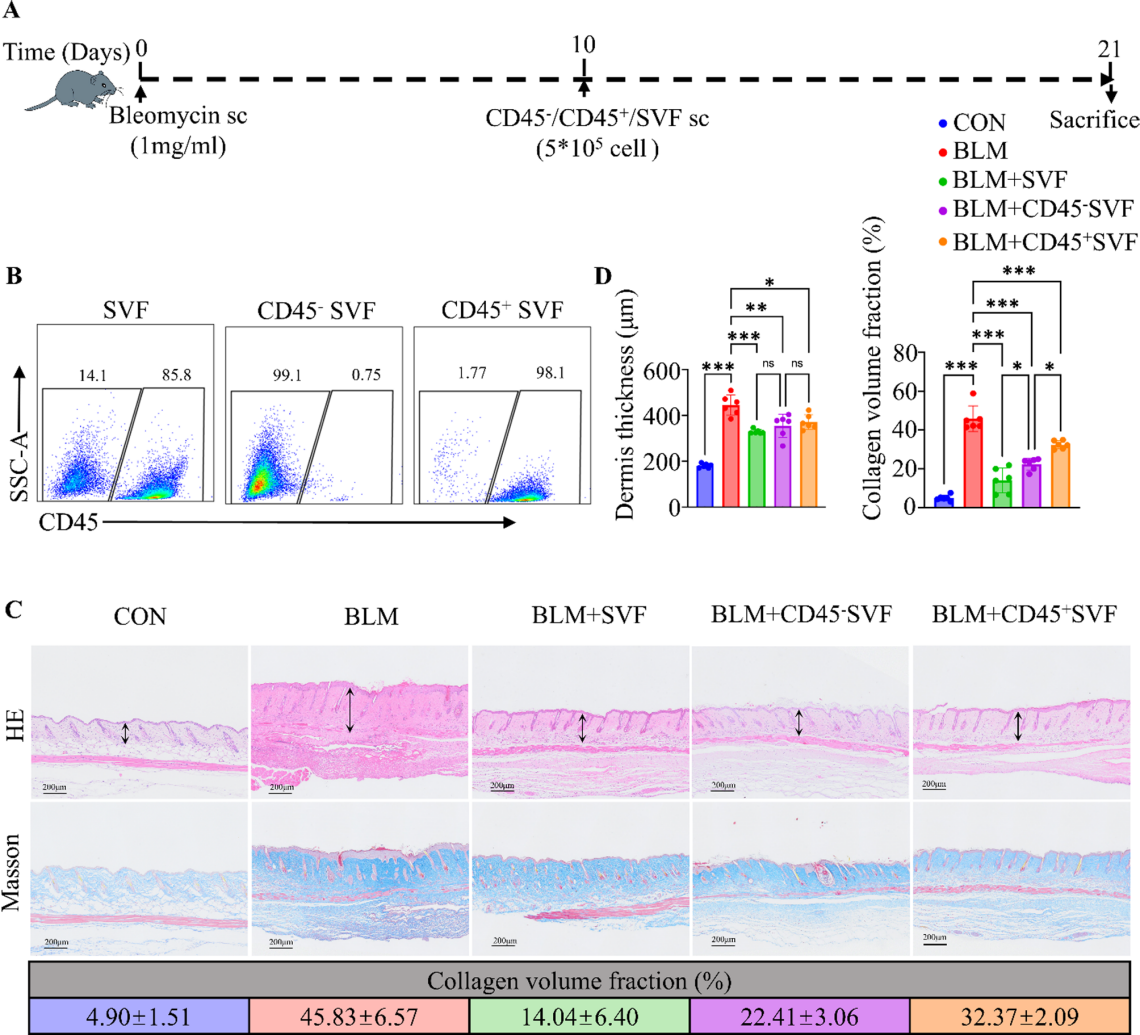
The CD45-negative subpopulation of SVF shows superior therapeutic effects compared with the CD45-positive subpopulation. **A**: Skin fibrosis was induced by subcutaneous injection of bleomycin in C57BL/6 mice; Dir-labeled SVF was administered subcutaneously on day 10 and assayed on days 14 and 21 post bleomycin modeling. **B**: Flow cytometric confirmation of the CD45-negative and CD45-positive subpopulations following magnetic bead separation. **C**: Representative hematoxylin and eosin (HE) and Masson’s trichrome images of skin from D21 fibrotic mice. Collagen volume fraction (%) is shown as mean ± SD (*n* = 6). **D**: Histograms of dermal thickness and collagen content in the late SVF administration groups (*n* = 6), analyzed by two-way ANOVA. Data are displayed as mean ± SD. *p < 0.05, **p < 0.01, ***p < 0.001

Using magnetic bead sorting to separate CD45-positive and CD45-negative cells within SVF (Fig. 6B), we administered subcutaneous injections of unsorted SVF, CD45-negative SVF, and CD45-positive SVF during the advanced fibrosis stage in a bleomycin-induced mouse model (Fig. 6A). All three interventions reduced dermal thickness and collagen deposition (Fig. 6C). Notably, the CD45-negative subpopulation exhibited antifibrotic efficacy similar to that of unsorted SVF in diminishing dermal thickness. Although SVF overall demonstrated the most robust inhibition of collagen deposition, the CD45-negative subpopulation was significantly more effective at reducing collagen content than the CD45-positive subpopulation (Fig. 6D). These findings indicate that the CD45-negative fraction of SVF can independently regulate skin fibrosis in advanced disease stages, outperforming the CD45-positive fraction. Consequently, CD45-negative cells appear to be the principal functional group within SVF responsible for its antifibrotic effects. These data provide a critical basis for optimizing SVF’s cellular composition and advancing its clinical applications.

## Discussion

In this study, we systematically evaluated the therapeutic efficacy of SVF in a bleomycin-induced mouse model of skin fibrosis, elucidated its mechanisms of action across different disease stages, and clarified the roles of its major functional subpopulations. These findings offer new perspectives on the clinical application of SVF in SSc-related skin fibrosis.

SVF is a heterogeneous cell population comprising immune cells, endothelial cells, and stromal cells. In vitro analyses demonstrated that SVF exhibits mesenchymal stem cell (MSC)-like properties and possesses multidirectional differentiation potential following adherent culture. Compared with traditional MSCs, SVF does not require extensive in vitro expansion, thereby preserving its native cellular microenvironment and paracrine functions—key advantages for clinical translation. Furthermore, SVF encompasses multiple functional cell types that can act synergistically via diverse mechanisms, potentially enhancing its therapeutic effects. Although clinical and preclinical research has examined SVF for osteoarthritis, wound healing, and fat grafting, investigations in fibrotic diseases have been comparatively limited. One previous study using a bleomycin-induced skin fibrosis model in nude mice showed that human SVF significantly reduces dermal thickness [22]. However, these data were restricted to a single time point and administration method, and they did not fully elucidate how different administration modes or durations of SVF treatment impact skin fibrosis and its underlying mechanisms. Moreover, nude mice lack a fully functional immune system, limiting the capacity of such models to accurately reflect SSc-associated skin fibrosis.

In the present study, we systematically analyzed the therapeutic potential of mouse-derived SVF in a bleomycin-induced skin fibrosis model of C57BL/6 mice, administering SVF at different disease stages and through distinct delivery routes. First, we demonstrated that early SVF administration, during the inflammatory phase, significantly inhibited bleomycin-induced inflammatory cell infiltration, dermal thickening, and collagen deposition. Second, we showed that SVF continued to exert notable antifibrotic effects when administered at the advanced fibrosis stage, with both subcutaneous and intradermal injections improving fibrosis; subcutaneous administration was slightly more effective. These findings suggest that SVF exerts consistent antifibrotic activity regardless of disease stage and underscore the importance of administration mode. Subcutaneous injection, as a minimally invasive approach, may be advantageous for clinical translation.

Further analyses revealed that SVF remains in fibrotic skin for prolonged periods. In vivo imaging and tissue section studies showed that SVF primarily persists in the subcutaneous layer rather than migrating into the dermis, implying that paracrine signaling, rather than direct differentiation, is the primary driver of its therapeutic effects. Additionally, the composition of SVF shifted dynamically over time, with the CD45-negative subpopulation becoming increasingly predominant. This observation suggests that the time-dependent redistribution of SVF cell subtypes may be central to its sustained antifibrotic efficacy.

When examining the mechanisms underlying SVF’s effects at early disease stages, we observed a significant increase in endothelial cells, accompanied by suppression of EndoMT in the dermal vasculature and a marked reduction in endothelial inflammation-related gene expression. These findings suggest that endothelial cells in SVF can facilitate vascular repair via paracrine mechanisms, thereby improving the early fibrotic microenvironment and enhancing long-term repair outcomes. Conversely, in late-stage administration, the proportion of endothelial cells did not increase substantially; instead, stromal cells became more dominant. This shift correlated with the secretion of antifibrotic factors such as HGF and basic FGF-2. HGF is known to mitigate inflammatory and fibrotic markers in skin fibrosis [23] and has been reported to be upregulated in fibrotic mouse skin following adipose-derived MSC therapy [24]. Our results confirm that SVF can directly release HGF subcutaneously in fibrotic mice, reinforcing the idea that stromal cells within SVF alleviate advanced fibrosis through paracrine mechanisms. Taken together, these findings show that SVF, as a heterogeneous cell population, exerts antifibrotic effects via distinct subpopulations at different pathological stages of skin fibrosis. This research advances our understanding of SVF’s therapeutic mechanisms and highlights its potential for targeted clinical interventions in SSc-related skin fibrosis.

Finally, by isolating CD45-negative and CD45-positive subpopulations, we confirmed the central role of the CD45-negative fraction in antifibrotic therapy. Specifically, the CD45-negative subpopulation alone produced therapeutic effects on skin fibrosis comparable to those of the intact SVF and outperformed the CD45-positive subpopulation. This observation underscores that CD45-negative cells are the primary functional group responsible for SVF’s antifibrotic effects, offering guidance for optimizing the cellular composition of SVF to enhance therapeutic efficacy. Notably, while certain studies have suggested that MSCs can adopt a fibroblast-like phenotype and exacerbate cardiac fibrosis in cardiac models [25], we found no evidence that CD45-negative SVF cells promote skin fibrosis. The discrepancy may be related to phenotypic differences between in vitro-cultured MSCs and those in SVF, as well as the distinct tissue microenvironments encountered by subcutaneously administered SVF in the skin compared to the heart.

Although the CD45-negative subpopulation emerged as a major therapeutic driver, the CD45-positive fraction may also contribute to the overall therapeutic benefit. In both the early and late phases of administration, the retained CD45-positive cells largely comprised macrophages. However, we could not distinguish whether the Dir-positive macrophages originated from the labeled SVF or if resident macrophages acquired Dir positivity through phagocytosing labeled SVF. Nevertheless, macrophages in the CD45-positive fraction may play a role in modulating skin fibrosis. Prior research has demonstrated that CD11b-positive cells in SVF modulate inflammation in gastrointestinal models [26, 27], implying that monocytes/macrophages in SVF could likewise ameliorate skin fibrosis by attenuating inflammation. Further investigation is warranted to determine whether macrophages can independently mitigate fibrosis and to explore potential interactions among SVF subpopulations.

Additionally, all SVF samples in this study were derived from healthy mice, whereas clinical interventions typically involve autologous SVF, which may have distinct immunological and cellular characteristics in patients with SSc. Nevertheless, some studies have reported that SVF from scleroderma patients exhibits proangiogenic effects in fibrotic mouse models similar to those observed with healthy-derived SVF [28]. Whether SVF derived from a fibrotic microenvironment demonstrates identical therapeutic efficacy and mechanisms remains to be determined. Consequently, investigating functional subpopulations and their mechanistic actions at different disease stages is of great importance. Standardizing SVF preparation and optimizing the purification of functional subpopulations for specific disease phases will be crucial for expediting the clinical translation of SVF-based therapies for fibrotic diseases.

In summary, this study demonstrates the substantial efficacy of SVF in treating skin fibrosis and clarifies the contributions of its major functional subpopulations. These findings provide a theoretical rationale for employing SVF in SSc-associated skin fibrosis, propose a novel therapeutic strategy, and offer new insights into managing other fibrosis-related conditions.

## Conclusion

This study shows that SVF has significant therapeutic potential for treating skin fibrosis related to SSc. We provide evidence that SVF effectively attenuates fibrosis during both early and late disease stages through distinct mechanisms. In the early phase, SVF alleviates skin vascular abnormalities and endothelial–mesenchymal transition, thereby preserving vascular integrity. In the advanced phase, SVF secretes antifibrotic factors such as HGF and basic FGF-2, reducing collagen deposition and dermal thickness. Notably, our findings highlight the pivotal role of the CD45-negative subset in mediating SVF’s antifibrotic effects, offering a direction for the development of more targeted cell-based therapies. Overall, SVF presents a promising treatment strategy for SSc-related skin fibrosis. Elucidating its mechanism of action could guide its application to a wider range of fibrotic diseases. Future studies should focus on refining SVF preparation and purification to facilitate clinical translation.

## Data Availability

The data used and analyzed during the current study is available from the corresponding author upon reasonable request.

## Abbreviations

SSc: Systemic sclerosis
SVF: Stromal vascular fraction
MSCs: Mesenchymal stem cells
HGF: Hepatocyte growth factor
FGF-2: Basic fibroblast growth factor
EndoMT: Endothelial-mesenchymal transition
